# Neurophysiological correlates of relatively enhanced local visual search in autistic adolescents

**DOI:** 10.1016/j.neuroimage.2006.11.036

**Published:** 2007-03

**Authors:** Zina M. Manjaly, Nicole Bruning, Susanne Neufang, Klaas E. Stephan, Sarah Brieber, John C. Marshall, Inge Kamp-Becker, Helmut Remschmidt, Beate Herpertz-Dahlmann, Kerstin Konrad, Gereon R. Fink

**Affiliations:** a Institute of Neuroscience and Biophysics, Department of Medicine, Research Centre Jülich, 52425 Jülich, Germany; b Brain Imaging Centre West, Research Center Jülich, 52425 Jülich, Germany; c Department of Child and Adolescent Psychiatry, University Hospital Aachen, 52074 Aachen, Germany; d Wellcome Trust Centre for Neuroimaging, Institute of Neurology, University College London, 12 Queen Square, London WC1N 3BG, UK; e Neuropsychology Unit, University Department of Clinical Neurology, Radcliffe Infirmary, OX2 6HE, Oxford, UK; f Department of Child and Adolescent Psychiatry, Philipps University Marburg, 35032 Marburg, Germany; g Department of Neurology, University of Cologne, 50931 Cologne, Germany

**Keywords:** Autism, Embedded Figures Task, Weak central coherence, Enhanced perceptual functioning, Primary visual cortex, Functional magnetic resonance imaging

## Abstract

Previous studies found normal or even superior performance of autistic patients on visuospatial tasks requiring local search, like the Embedded Figures Task (EFT). A well-known interpretation of this is “weak central coherence”, i.e. autistic patients may show a reduced general ability to process information in its context and may therefore have a tendency to favour local over global aspects of information processing. An alternative view is that the local processing advantage in the EFT may result from a relative amplification of early perceptual processes which boosts processing of local stimulus properties but does not affect processing of global context. This study used functional magnetic resonance imaging (fMRI) in 12 autistic adolescents (9 Asperger and 3 high-functioning autistic patients) and 12 matched controls to help distinguish, on neurophysiological grounds, between these two accounts of EFT performance in autistic patients. Behaviourally, we found autistic individuals to be unimpaired during the EFT while they were significantly worse at performing a closely matched control task with minimal local search requirements. The fMRI results showed that activations specific for the local search aspects of the EFT were left-lateralised in parietal and premotor areas for the control group (as previously demonstrated for adults), whereas for the patients these activations were found in right primary visual cortex and bilateral extrastriate areas. These results suggest that enhanced local processing in early visual areas, as opposed to impaired processing of global context, is characteristic for performance of the EFT by autistic patients.

## Introduction

**Fig. 1:**
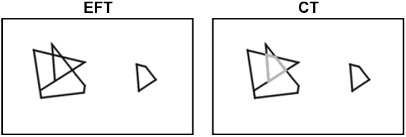
Two examples of the stimuli used for the Embedded Figures Task (EFT) and the control task (CT).

Autism is a developmental disorder characterized by impaired social interaction and communication as well as by repetitive behaviours and restricted general interests. Over the last decades, several theories have been developed that attempt to explain the cause of autism in terms of dysfunctional central cognitive processes. In addition to theories of impairments in Theory of Mind (Baron-Cohen et al., 2000; Happé, 1994) and executive dysfunction (Ozonoff et al., 1991), substantial interest has been devoted to perceptual abnormalities in autism (Behrmann et al., 2006). For example, an influential finding in autism research was that autistic patients tend to show superiority for tasks where local processing strategies are beneficial. A classical example of this is the Embedded Figures Task (EFT). Originally devised by Gottschaldt (1926), the EFT involves search for a target figure hidden in complex visual pattern (see Fig. 1 for an example). Subjects are required to decide as quickly as possible whether or not the simpler target shape is “hidden” in the complex figure. Shah and Frith (1983) were the first to discover that autistic children responded both faster and more accurately on the EFT compared to matched control children. The initial findings by Shah and Frith (1983) were subsequently replicated by Jolliffe and Baron-Cohen (1997) who found both autistic and Asperger children to perform better on the EFT than controls. Other studies found non-significant differences in the behavioural performance of patients and controls on the EFT (Brian and Bryson, 1996; Ring et al., 1999; Ropar and Mitchell, 2001), but even this is remarkable given that autistic patients usually tend to perform worse than controls on most complex cognitive tasks. This behavioural advantage (absolute or relative, depending on the study) of autistic patients on the EFT has been interpreted in two major ways: (i) in terms of “weak central coherence” (WCC; Frith, 1989; Shah and Frith, 1983; Happé, 1999; Hill and Frith, 2003; Jolliffe and Baron-Cohen, 1997) and (ii) from the perspective of theories that postulate enhancements of early perceptual processes in autism (Mottron et al., 2006; Plaisted et al., 2003).

Central coherence describes the ability to integrate separate pieces of information into meaningful wholes. In relation to autism, the WCC theory postulates a general (domain-unspecific) tendency to favour processing of local stimulus properties due to a reduced ability in processing global context (Frith, 1989; Happé, 1999). It assumes that WCC occurs at both “low” and “high” levels of information processing. Low-level WCC refers to the tendency to neglect context in the sensory (e.g. visual) domain, favouring the processing of individual stimulus features, whereas high-level WCC concerns impairments of more abstract contextual processes (Happé, 1996a,b; Jolliffe and Baron-Cohen, 1997). WCC is not necessarily always a cognitive limitation but should actually be advantageous for perceptual tasks which require processing of local aspects of complex stimuli, like the EFT (Shah and Frith, 1983; Happé, 1999).

According to the classical WCC theory, superior performance of autistic patients in local processing during the EFT results from a deficiency of global context processing. However, some studies using hierarchically structured stimuli suggested that autistic patients respond to the global stimulus level with similar efficiency as controls (Mottron et al., 1999, 2003; Ozonoff et al., 1994; Plaisted et al., 1999, 2003). These findings imply that autistic patients might not necessarily show a deficiency in global context processing with resulting superior local processing but could instead have a primary superiority in local processing, with global processing being unaffected. Such a perspective has been formulated with a strong focus on perception, most prominently by Plaisted and colleagues (Plaisted et al., 1998, 2003; O’Riordan and Plaisted, 2001) and Mottron and Burack (Mottron and Burack, 2001; Mottron et al., 2006). Plaisted and colleagues have suggested that atypical perceptual processes in autism enhance the salience of individual stimulus features without compromising global processing. Their experiments suggested that this enhancement occurs at very early stages of sensory processing hierarchies (Plaisted et al., 1998, 2003; O’Riordan and Plaisted, 2001). A related, although not identical, account of abnormal perception in autism is provided by the Enhanced Perceptual Functioning (EPF) theory (Mottron and Burack, 2001; Mottron et al., 2006). According to this theory, low-level perceptual processing is abnormally enhanced relative to high-level cognitive processes, making automatic perceptual processes more difficult to control by top-down mechanisms and thus more likely to supersede or interfere with higher cognitive processes. Neurophysiologically, this abnormality is proposed to be reflected by a general “skewing” of brain activation towards primary sensory areas in autistic patients, providing the basis of a superior "perceptual trace" that enhances memory for local stimulus properties and thus might explain the good performance of autistic patients on the EFT (Mottron et al., 2006).

So far, there is little neurophysiological evidence that would help distinguishing between the WCC theory and its alternatives. For each theory, predictions can be derived concerning the differences in brain activity between a task requiring local visual search (like the EFT) and a similar control task with reduced local search requirements. Notwithstanding their subtle differences, the theories by Plaisted and Mottron and Burack, respectively, both postulate that early visual areas, whose small receptive field sizes are appropriate for perceptual processing of local stimulus properties, should show higher activation in patients compared to controls. For the classical WCC theory, precise predictions about the sites of differential activations during the EFT have not been formulated in detail before but can be derived on the basis of recent studies. It is well established that several left-hemispheric areas beyond extrastriate cortex like the IPS (Weissman and Woldorff, 2005) are crucial for processing local stimulus properties, despite relatively large receptive fields. In contrast, global stimulus properties are predominantly processed by right-hemispheric areas (Hellige, 1996; Martinez et al., 1997; Proverbio et al., 1998; Robertson and Lamb, 1991; Weissman and Woldorff, 2005; Yamaguchi et al., 2000). For early visual areas with small receptive fields (see above), such a lateralisation is more controversial (Fink et al., 1996, 1997; Sasaki et al., 2001; Weissman and Woldorff, 2005). Therefore, contrasting a task with strong local processing requirements like the EFT to a control task with similar stimuli but minimal local processing requirements, one would expect left-lateralised activations which may or may not include early visual cortices, but should definitely be observed for “higher” areas, particularly the IPS (Weissman and Woldorff, 2005). This is exactly what we found in a previous study of adults where the local processing requirements of the EFT activated the left IPS and the left inferior frontal gyrus (IFG; Manjaly et al., 2003). Critically, since the WCC theory assumes deficient global processing, it would predict attenuated right-hemispheric activity and that the lateralisation of activity during the EFT to left IPS and left IFG should be more pronounced in autistic subjects than in controls. So far, the only neuroimaging study of the EFT in autism is an fMRI study by Ring et al. (1999). Unfortunately, this study had a small sample size (six patients) and only compared EFT against rest (fixation), therefore the activations are relatively unspecific and not easily interpretable.

The present study aimed at providing more specific neurophysiological evidence to distinguish between the different theories of autism. Our recently established EFT paradigm (Manjaly et al., 2003) contrasts the EFT with a closely matched visuospatial control task (CT) with very similar stimuli but minimal local search requirements, as well as with a simple shape recognition task. This paradigm was combined with fMRI to measure brain activity in age- and IQ-matched groups of children with autistic spectrum disorders and control children in order to determine whether the EFT-specific activations would support the WCC theory or the perceptual theories by Plaisted and Mottron and Burack, respectively.

## Materials and methods

### Subjects

12 right handed adolescents with the diagnosis of Asperger syndrome (*n* = 9) or High-Functioning Autism (HFA; *n* = 3) and a mean age of 14.4 years (SD = 2.7 years) were studied in comparison to 12 controls (14.3 ± 2.7 years) without any history of neurological or psychiatric illness. Since the majority of studies suggest that both, HFA and Asperger syndrome, belong to the same spectrum disorder (Gilchrist et al., 2001; Frith and Happé, 2005), we decided to include either patients with HFA or Asperger syndrome in the autistic group. The two groups were matched for gender, age, handedness, and IQ as measured with the Culture Fair Intelligence Test 20 (CFT 20, Weiß, 1998). All children and their parents or caregivers gave their written informed consent after having been informed about the details and the purpose of this study. The study was approved by the ethics committee of the University Hospital Aachen.

The autistic children were diagnosed by experienced clinicians according to the standard criteria of ICD-10 (World Health Organization, 1993) and DSM-IV (American Psychiatric Association, 1994) and underwent extensive psychiatric examination. The expression of autistic symptoms was further assessed by the German version of the Autism Diagnostic Observation Scale (ADOS-G; Lord et al., 2000) and a semi-structured autism specific parent interview (ADI-R; Le Couteur et al. 1989); this in-depth assessment was conducted by trained examiners (N.B., I. K.-B.). Subjects with Asperger syndrome had an IQ above 80, met DSM-IV criteria for Asperger syndrome or autism and fulfilled the cut-off criteria of the ADOS-G and ADI-R for autism or autism spectrum disorder. Subjects with HFA fulfilled ADI-R and ADOS-G threshold scores for autism, but had an IQ of at least 80, and a history of phrase speech development at 36 months or older. Since many persons with Asperger syndrome also meet ADI-R and DSM-IV criteria for autism (e.g. Mayes et al., 2001) the primary distinguishing feature between individuals with HFA and Asperger syndrome was a history of clinically significant language impairment (see also Howlin, 2003). Additionally, all parents of the autistic group completed the German version of the Autism Screening Questionnaire (ASQ; Boelte et al., 2000). To exclude clinically relevant psychopathology, the Child Behaviour Checklist (CBCL; Achenbach and Edelbrock, 1983) was completed by all parents in both groups. None of the subjects showed any relevant somatic or psychiatric disorder except autism in the clinical group. Table 1 summarizes the major clinical and demographic data. To minimize movement artefacts and to prevent anxiety in the unfamiliar surroundings, all subjects were familiarized to the fMRI scanner environment using a simulated (“mock”) MRI scanner that looked and sounded similar to the real scanner.

### Stimuli and experimental design

In a previous study (Manjaly et al., 2003), we established an fMRI-compatible EFT version for adults. This version turned out to be too difficult and too long for young adolescents, particularly patients. For the present study, we therefore adapted our version of the EFT. Using a PC graphics program (Corel Draw 9.0, Corel Inc.), 12 figures of comparable complexity were created that consisted of the same number of lines (8). Each of these stimuli was presented eight times in the experiment, four times in the EFT condition and four times in the control task (CT, see below), resulting in 48 trials per condition. On each trial, a target figure was displayed next to the complex figure (50% left, 50% right). In 50% of all trials the accompanying target figure was embedded in the complex figure (“correct” target figure) and in 50% it was not (“incorrect” target figure). To prevent priming effects, each correct target figure was only presented once throughout the experiment. Because the incorrect target figures were not embedded in any complex figure shown in the experiment, they could safely be used twice, i.e. once each in EFT and CT. Fig. 1 shows examples of the stimuli used in the EFT and the CT.

In the EFT, subjects had to decide whether or not the target figure matched any subpart of the complex figure. Crucially, the EFT requires one to dissect the complex figure into local substructures in order to decide whether any of them matches the target figure. To control for other cognitive processes involved in the EFT, e.g. more general aspects of visual search and perception of complex geometric figures, we used a control task (CT) which comprised all cognitive aspects of the EFT but had minimal local visual search requirements. In this CT, a substructure of the complex figure was highlighted with a grey line, and subjects had to decide if the outlined substructure was the same as the simultaneously presented target figure or not. As a further control, we used a high-level baseline (BL) in which subjects were presented with a triangle or a square and had to discriminate between them. This shape recognition task controlled for aspects common to both EFT and CT, i.e. visual recognition and attention, decision-making and motor responses, but did not involve any visuospatial search or matching processes. Overall, our paradigm thus had a hierarchical structure, with the CT controlling for all aspects of the EFT except the significant local visual search component, and the BL controlling for more basic cognitive and motor processes present in both EFT and CT. In our previous study (Manjaly et al., 2003), stimuli during EFT and CT were presented either horizontally or vertically. Since we did not find any significant task-by-orientation interaction in that previous experiment, we only used a horizontal arrangement of stimuli in the present study.

All answers were recorded via button presses, using MRI-compatible key pads. Stimuli were presented in black on a white background on a 30 × 15 cm screen (horizontal visual angle of 42.3°, vertical visual angle of 24.4°) for 3000 ms (stimulus on-time, SOT) with an inter-stimulus interval (ISI) of 1000 ms (blank screen) using Presentation 7.96 (Neurobehavioural Systems Inc., San Francisco). SOT and ISI were identical for all conditions. Subjects viewed the display from a distance of 33 cm (20 cm screen to mirror, 13 cm mirror to subjects’ eyes). The complex figure and target figure combined covered a visual angle of 16.9° horizontally and 3.5° vertically (combined stimulus width 10 cm, height 2 cm). In 50% of the trials the target appeared left and the complex figure appeared right ; in the other 50% of the trials these positions were reversed. We controlled for order effects by (i) counterbalancing the condition order across subjects and by (ii) randomizing the order of stimuli across blocks and conditions for each subject.

A blocked design was adopted in order to maximize statistical efficiency. Blocks from all conditions consisted of 6 trials, and each block lasted 24 s, preceded by a short instruction screen (6 s). The experiment consisted of 8 EFT blocks, 8 CT blocks, and 16 BL blocks, giving a total scanning time of 16 min.

Reaction times and error rates were recorded as behavioural measures. Subjects were instructed to respond as quickly and as accurately as possible in all tasks. In all conditions, the number of required “yes” and “no” responses was 50% each. The subjects’ condition-specific mean reaction times and mean error rates of all two experimental runs were compared by analysis of variance (ANOVA), using SPSS V11 (SPSS Inc., Chicago, IL).

### MRI acquisition

Echo Planar Imaging was performed on a Siemens Sonata 1.5 T scanner using a standard head coil. Pulse sequence parameters were as follows: TE = 66 ms; TR = 3.02s; FOV = 200 × 200 mm; *θ* = 90°; matrix size = 64 × 64; pixel size = 3.125 × 3.125 mm; slice thickness = 4.0 mm; inter-slice gap = 0.4 mm; 30 slices. Additionally, we obtained high-resolution, T1-weighted structural brain images using a standard MP-RAGE (magnetisation-prepared, rapid acquisition gradient echo) sequence.

### fMRI data analysis

All calculations and image manipulations were performed on Sun Ultra 60 workstations (SUN Microsystems Computers) using MATLAB 6.5 (The Mathworks Inc., Natick, MA, USA) and SPM2 (Statistical Parametric Mapping, SPM; Wellcome Department of Imaging Neuroscience, London, UK).

For each subject, the first five images were discarded, the remaining 320 images were realigned to the first image to correct for head movements, spatially normalised, i.e. transformed into a standard stereotactic space as defined by the MNI template (Montreal Neurological Institute) using a non-linear warping algorithm (Ashburner and Friston, 1999), and resampled to a voxel size of 3 × 3 × 3 mm. The normalised scans from each subject were smoothed with a three-dimensional Gaussian kernel of 10 mm full width half maximum for the group analysis to meet the statistical requirements of the General Linear Model and to compensate for remaining inter-individual variability in anatomy across subjects.

Data were analysed using a general linear model in SPM2, removing subject-specific low frequency signal drifts by a high pass filter of 128 s The different conditions were modelled by convolving box car functions with a canonical haemodynamic response function. In addition to regressors modelling the three different tasks and the instruction periods, we included six regressors in the design matrix that consisted of the realignment parameters, describing rotation and translation of the subject’s head during the experiment. This allowed us to regress out any variance that could be explained by a linear combination of head translations and rotations. After estimation of the model parameters, specific effects were tested for by applying appropriate linear contrasts to the parameter estimates, resulting in contrast images. Specifically, we computed contrasts for each relevant task pair, i.e. EFT > CT, CT > EFT, EFT > BL, CT > BL.

Subsequently, contrast images were entered into second-level *t*-tests (one-sample *t*-tests for within-group analyses, two-sample *t*-tests for between-group analyses), implementing random effects group analyses (Penny and Holmes, 2004). These analyses, which used the non-sphericity correction of SPM2 to take into account potential group differences in variance, resulted in a *t*-statistic for each voxel. The significance of local topological features of the resulting SPM{T} can be determined using Gaussian random field theory. In all analyses, areas of activation were identified as significant only if they passed a threshold of *p* < 0.05, corrected for multiple comparisons at the cluster level (Poline et al., 1997), using a standard *p* < 0.001 cut-off at the voxel-level.

In addition to the specific contrasts of interest, we also tested for the overall effect of visual analysis of complex geometric figures with differing degrees of local search relative to pure recognition of geometric shapes. For this purpose, we performed a random effects conjunction analysis, based on inclusive masking, as suggested by Nichols et al. (2005). This conservative analysis corresponds to a logical AND operation, showing those voxels which are significant in both the EFT > BL and CT > BL comparisons in both patients and controls (Fig. 3). For this analysis we used a threshold of *p* < 0.05, corrected for multiple comparisons at the voxel level.

## Results

### Behavioural measures

We analysed both reaction times (RTs) and the percentage of correct (PC) responses across all volunteers using a two-factorial ANOVA with factors “task” (EFT vs. CT) and “group” (patients vs. controls) and Greenhouse–Geisser correction for non-sphericity. Reaction times were computed for correct responses only. The results are summarized by Table 2.

**Fig. 2:**
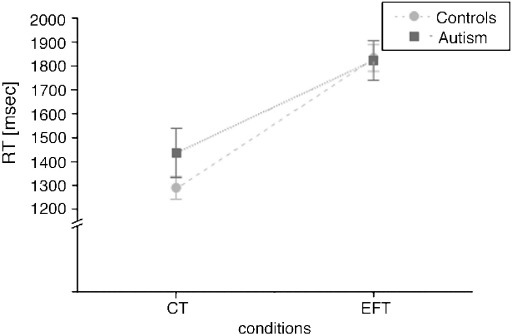
Mean reaction times (in ms) of both the patient and control group for EFT and CT. Error bars denote standard error. Patients and controls are almost identical in their reaction times in the EFT, whereas patients respond much more slowly than controls in the control task. This group × condition interaction was bordering significance (*p* < 0.054).

As expected, the analysis of RTs showed a main effect of task, i.e. pooled across groups responses were faster for the CT (1362 ± 57 ms [mean ± standard error]) than for the EFT (1829 ± 50 ms) (*p* < 0.001). In contrast, there was no main effect of group, i.e. pooled across tasks the RTs in the autistic group (1630 ± 86 ms) were not significantly different from the controls (1561 ± 50 ms) (*p* = 0.496). However, while there was virtually no difference between groups for the EFT (patients: 1825 ± 83 ms; controls: 1834 ± 56 ms; *p* = 0.9), patients took numerically longer to respond during the CT than the controls (patients: 1435 ± 102 ms; controls: 1288 ± 49 ms; *p* = 0.2) (see Fig. 2), and the resulting task-by-group interaction was at the borderline of significance (*p* = 0.054).

Analysing the percentage of correct responses (PC), again a significant main effect of task was found, i.e. pooled across groups subjects made more correct decisions for the CT (95.9 ± 1.4%) than for the EFT (87.8 ± 1.5%) (*p* < 0.001). There was no main effect of group, i.e. pooled across tasks the proportion of correct responses in the patient group (89.8 ± 2.3%) was not significantly different from that in the control group (93.8 ± 1.1%) (*p* = 0.126). In contrast to the RT data, there was no task-by-group interaction (*p* = 0.805).

### fMRI results

**Fig. 3:**
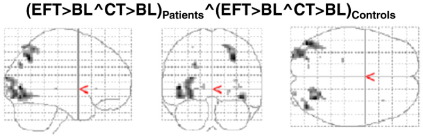
Random effects conjunction analysis, based on inclusive masking, which shows those clusters that are significant in both the EFT > BL and CT > BL comparisons, across both groups. This analysis tests for the overall effect of visual search in geometric figures (EFT and CT) relative to pure recognition of geometric shapes (baseline condition, BL), in both patients and controls. Results are thresholded at *p* < 0.05 at the voxel level, corrected for multiple comparisons across the whole brain.

Initially, we determined the areas that were activated, in both patients and controls, for visual processing of complex geometric figures as compared to simple shape recognition. For this purpose, we used a conjunction analysis that was based on inclusive masking of the contrasts EFT > BL and CT > BL (Nichols et al., 2005). This “logical AND” conjunction analysis revealed that both autistic subjects and control subjects showed activations, for contrasting both EFT and CT against BL, in the middle occipital gyrus and the intraparietal sulcus bilaterally, as well as activations in right fusiform gyrus, right inferior occipital gyrus, left lingual gyrus, left superior parietal gyrus, right anterior insula and left thalamus (*p* < 0.05, corrected; see Fig. 3 and Table 3 for coordinates of the maxima).

**Fig. 4:**
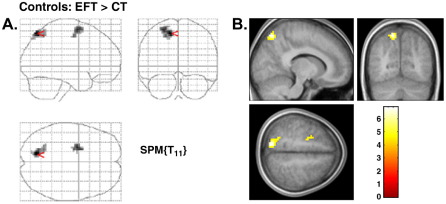
Random effects analysis of the EFT > CT contrast within the control group. This analysis tests for effects specific for the local visual search processes required by the EFT but not by the otherwise closely matched CT. (A) Maximum intensity projection of significant clusters. Results are thresholded at *p* < 0.001 and are cluster-level corrected for multiple comparisons across the whole brain at *p* < 0.05. (B) The SPM{T} overlaid on the mean structural image of the group. The colour bar indicates T-values. (For interpretation of the references to colour in this figure legend, the reader is referred to the web version of this article.)

More importantly, however, was the difference in activation during performance of the EFT in comparison to CT, separately in both groups. All results reported below are significant at the cluster-level (*p* < 0.05), corrected for multiple comparisons across the whole brain. The coordinates and *T*-values given below refer to the local maxima of the significant clusters. Assessing the EFT vs. CT contrast in the control group showed an activation in left posterior parietal cortex (maximum at *x* = − 12, *y* = − 72, z = 57; T = 6.88) and left dorsal premotor cortex (− 24,− 3, 63; *T* = 6.27; see Fig. 4 and Table 4). This activation pattern is a fairly good replication of our previous results when testing adult volunteers on the EFT (Manjaly et al., 2003); see Discussion. The reverse contrast, i.e. CT > EFT, demonstrated an activation in the left medial temporal lobe (− 27,− 18,− 18; *T* = 9.18).

**Fig. 5:**
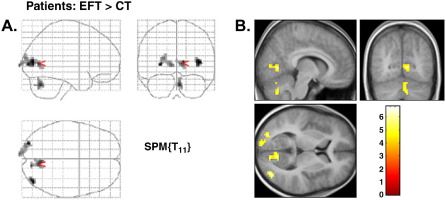
Random effects analysis of the EFT > CT contrast within the patient group. See Fig. 5 for explanations.

The group of autistic subjects revealed a different activation pattern (see Fig. 5 and Table 4). When comparing EFT to CT, significant activations were found in the left (− 27, − 90, 3; *T* = 6.51) and right (39, − 81, 9; *T* = 6.79) extrastriate cortex, in the cortex around the right calcarine sulcus (9, − 72, 6; *T* = 5.83), and in the right cerebellar hemisphere, extending into the vermis (6, − 69, − 33; *T* = 5.88). When comparing the locations of the activations in visual areas against a probabilistic cytoarchitectonic atlas (http://www.bic.mni.mcgill.ca/cytoarchitectonics) that was warped into MNI space (see Eickhoff et al., 2005 for the methodology), we found that the local maximum of the activation in the vicinity of the right calcarine sulcus (9, − 72, 6) had a high probability (80%) of being located in area V1. In contrast, the two local maxima of the activations in left and right extrastriate cortex had zero percent probability of being located in either V1 or V2. The reverse task comparison, i.e. CT > EFT, did not show any significant activation in the patient group.

**Fig. 6:**
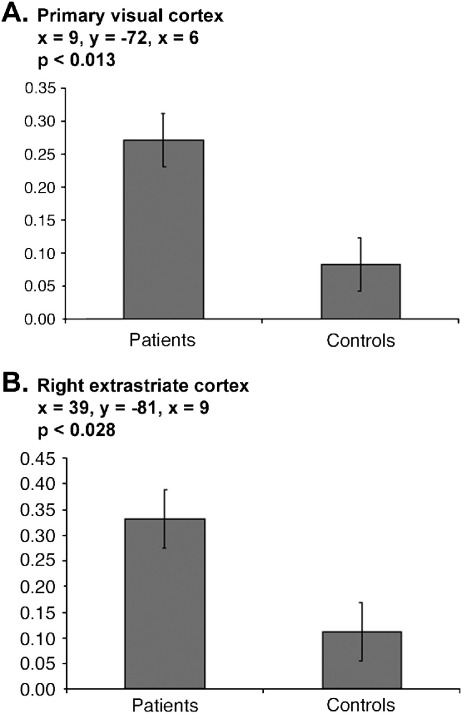
This bar plot shows the contrast value for the EFT > CT contrast for two regions selected from the analysis in Fig. 5, averaged across subjects. The values correspond to percent signal change, relative to global brain mean signal. Error bars denote standard error. Although the EFT vs. CT parameter difference is larger in the patient group compared to controls, and this difference is significant (*p* < 0.05) at the level of the individual voxels studied in primary visual (A) and right extrastriate cortex (B), it did not survive correction for multiple comparisons.

In a next step, we assessed whether there were any brain regions in which the difference between EFT and CT was significantly different between patients and controls. This corresponds to testing for group-by-condition interactions, which can be implemented by a two-sample *t*-test operating on the within-group contrast images. This analysis did not yield any results that were significant after correcting for multiple comparisons across the whole brain. However, at uncorrected levels of significance, some of the regions (e.g. calcarine sulcus and right extrastriate cortex) which were significantly activated in the EFT vs. CT contrast in the autistic group, also showed such an interaction, i.e. higher EFT vs. CT differences in the autistic than in the control group (see Fig. 6).

## Discussion

Compared to previous work, the present study is, to our knowledge, the first to simultaneously assess behavioural performance on an additional visual task (CT) that closely matches the EFT in all cognitive processes except the major local visual processing component itself. While our behavioural results do not support the notion that autistic patients have an *absolute* advantage for local visual processing, they do suggest that autistic individuals have a *relative* advantage for local processing. This was reflected by a task-by-group interaction (*p* = 0.054) in terms of RTs. This interaction indicated that autistic patients were equally fast on the EFT as the control subjects (Table 2; note that error rates were also very similar), but showed slower responses on the CT where local processing requirements were minimal. How does this finding fit with previous behavioural studies of EFT performance by autistic and control subjects? In the classical study by Shah and Frith (1983) as well as in the study by Jolliffe and Baron-Cohen (1997) autistic children were found to respond both faster and more accurately than control children. This superior performance of the autistic group was consistent with the findings of Kagan and Kogan (1970) who found an inverse relation between sensitivity to social cues and performance on the EFT in children. Similarly, Baron-Cohen and Hammer (1997) reported that parents of children with Asperger syndrome were significantly faster on the EFT than a control group of parents with non-autistic children. Several other studies provided additional evidence for better performance of autistic patients in detecting embedded figures (de Jonge et al., 2006; Jarrold et al., 2005; Pellicano et al., 2006). Two other studies, however, deviated from this pattern. Ropar and Mitchell (2001) found that autistic and Asperger children performed equally well on the EFT as control children. A study by Brian and Bryson (1996) also failed to find superior performance of autistic patients compared to controls. The latter study, however, is difficult to interpret because the groups differed in age.

The current study achieved a fairly good replication of our previous fMRI results of adult volunteers performing the EFT (Manjaly et al., 2003). In that study contrasting EFT to CT revealed significant activations in the left posterior parietal cortex, including the IPS, and in the left ventral premotor cortex (posterior IFG). This finding is consistent with the well-established left-hemispheric dominance for local visual processing (see Introduction). Using a probabilistic cytoarchitectonic atlas, the premotor activation was found to overlap with areas 44 and area 6. A subsequent connectivity analysis demonstrated that this activation functionally interacted with areas commonly involved in visuospatial processing (Manjaly et al., 2005). In the present study, comparing EFT to CT in the control group activated the left IPS and the left dorsal premotor cortex. While the parietal activation reported here was very similar to the one found by Manjaly et al. (2003), the premotor activation was more dorsally located. This may be due to differences in local processing in adults and adolescents, or it may result from differences in the design of the two studies. For example, the stimuli used by the current study were less complex (eight instead of twelve composing lines) and were shown in horizontal orientation only.

Importantly, the patient group showed a different activation pattern when comparing EFT to CT. Here, we did not find a further increase in the left-lateralisation of activity in IPS and other “higher” areas as implied by the classical WCC theory (see Introduction). Instead, activations were found in the cortex surrounding the right calcarine sulcus and in the extrastriate cortex bilaterally. These activations at the early stages of visual processing in autistic individuals are compatible with the hypotheses by Plaisted et al. and Mottron and Burack, respectively, that the advantage of autistic patients for local visual processing may be due to differences in basic perceptual processes. According to Plaisted et al., in the early sensory cortices of autistic patients the salience of individual stimulus features is boosted without compromising processes of global integration (Plaisted et al., 1998, 2003; O’Riordan and Plaisted, 2001). Similarly, the EPF theory by Mottron and Burack holds that superior perceptual processing and enhanced “perceptual traces” in early visual cortex could induce better memory for local properties of visual stimuli (Mottron and Burack, 2001). In a recent review of the evidence for this, Mottron et al. (2006) suggested that enhanced V1 activation may be a general phenomenon for visual tasks in autism. Finally, we also found an activation in the right cerebellar hemisphere, extending into the vermis, which is neither predicted by the WCC theory nor by the other theories. Currently, we cannot offer a good explanation for this finding.

The direct group comparison between patients and controls for the EFT vs. CT contrast did not yield any brain regions with between-group differences in local processing that were significant at *p* < 0.05 corrected. However, at uncorrected levels of significance, some of the regions (e.g. the calcarine sulcus) which were significantly activated in the EFT vs. CT contrast in the autistic group, also showed a task × group interaction, i.e. higher EFT vs. CT differences in the autistic group compared to the control group (see Fig. 6).

One limitation of our study is the restricted sample size. Even though we studied twice as many patients (12) than the only other EFT fMRI study in autism available so far (Ring et al., 1999), this is still a limited number given recent evidence that there may be considerable variance with regard to EFT performance among patients with autistic spectrum disorder (Edgin and Pennington, 2005). Ideally, one would subdivide the patients into subgroups with different performance levels on the EFT. With the current sample size, however, this was not feasible statistically. This approach is suggested for future studies with larger sample sizes, and the present findings should be treated as preliminary results until confirmed in larger samples.

Notwithstanding the above caveats, our present results, which point to differential information processing at early stages of visual perception in autism, are in line with evidence from a growing body of psychophysical, electrophysiological and fMRI studies (reviewed by Plaisted et al., 2003; Mottron et al., 2006). For example, the psychophysical experiments by Bertone et al. (2005) and Caron et al. (2006) pointed to differences in processing orientation information and perceptual cohesiveness of visual stimuli, respectively, at the level of early visual areas in autistic patients. McPartland et al. (2004) demonstrated an abnormal N170 event-related potential at early stages of face processing. Brown et al. (2005) reported abnormal EEG gamma activity over visual cortex, possibly reflecting a diminished signal-to-noise ratio due to decreased inhibitory processing at the early stages of perception in autism. Koshino et al. (2005) reported increased activity in extrastriate areas of autistic patients, compared to controls, during a visual N-back working memory task studied with fMRI. Belmonte and Yurgelun-Todd (2003) applied fMRI to a simple visuospatial attention task and found activations in primary visual and ventral occipital cortex of autistic subjects, but not of controls.

Concerning local visual search in particular, so far only one previous study has examined the neural mechanisms underlying local visual processing in autistic patients (Ring et al., 1999). This fMRI study investigated six autistic subjects and twelve controls, contrasting the EFT with an unspecific ‘fixation only’ condition. When comparing the EFT-related activations between groups, Ring et al. found that the only regions exhibiting higher activity in the autistic group were primary and secondary visual areas. The far more specific EFT vs. CT contrast in our study produced results for the autistic group that are compatible with the findings by Ring et al. (1999).

The present study included a carefully designed control condition (CT) for a task probing local visual processing (EFT). However, it should be noted that while this task controlled for all motor, cognitive, and perceptual processes (except local visual search) of the EFT, it did not directly probe the capacity for global integration. A next step therefore is to directly compare with fMRI local and global processing capacities in larger samples of autistic patients, e.g. using identical, hierarchically structured stimuli (Rinehart et al., 2000; Fink et al., 1997). In addition, future studies should focus on the direct comparison between different childhood psychiatric disorders characterized by attentional problems in order to clarify how specific these cognitive profiles are for patients with autism.

## Figures and Tables

**Table 1:** Demographic and clinical data of the subjects in this study

	Controls	Autism	*p*
Mean (range; SD)	Mean (range; SD)
*N*	12	12	
Age (years)	14.3 (10–18; 2.7)	14.4 (10–17; 2.8)	> 0.94
IQ (CFT-20)	109.3 (83–132; 13.6)	110.1 (83–134; 20.0)	> 0.92
Handedness	12 right-handed	12 right-handed	
DSM-IV/ ICD-10 Diagnosis			
Asperger syndrome	0	9	
High-functioning Autism (HFA)	0	3	

**Table 2:** Behavioural results of both groups on the Embedded Figures Task (EFT), Control Task (T) and the baseline condition (BL)

	Patients (*N* = 12)			Controls (*N* = 12)		
Mean	SE	Range	Mean	SE	Range
RT BL [ms]	683	99	439–1559	694	51	448–1032
RT CT [ms]	1435	102	993–2236	1288	49	1119–1661
RT EFT [ms]	1825	83	1331–2188	1834	56	1545–2222
% correct CT	94.1	2.6	68.75–100	97.7	0.8	91.67–100
% correct EFT	85.6	2.5	68.75–93.75	89.9	1.6	79.17–95.83

**Table 3:** Conjunction analysis (EFT > BL AND CT > BL)_Patients_ AND  (EFT > BL AND CT > BL)_Controls_ of the fMRI data

	Side	*x*	*y*	*z*	*k*
Middle occipital gyrus	left	− 45	− 78	3	173
	right	24	− 93	− 3	4
	right	33	− 84	12	4
	right	30	− 99	3	1
Intraparietal sulcus	right	30	− 66	42	72
	left	− 24	− 78	33	10
Fusiform gyrus	right	39	− 78	− 12	33
Inferior occipital gyrus	right	39	− 93	3	4
Lingual gyrus	left	− 12	− 75	3	4
Thalamus	left	− 24	− 30	0	2
Superior parietal gyrus	left	− 27	− 60	57	1
Anterior insula	right	33	24	0	1

This table lists the results from the conjunction analysis (implemented by inclusive masking) shown by Fig. 3. All results are significant at *p* < 0.05 at the voxel level, corrected for multiple comparisons across the whole brain. Note that because of the multiple masking involved in this analysis, it is not meaningful to list individual *T* scores. *k* = number of activated voxels; *x*, *y*, *z* = coordinates of local maxima in MNI space.

**Table 4:** fMRI results: EFT > CT, separately in the autistic and the control group

	Side	*x*	*y*	*z*	T score	*k*
*Controls*						
Posterior parietal cortex	left	− 12	− 72	57	6.88	86
Dorsal premotor cortex	left	− 24	− 3	63	6.27	67

*Autistic*						
Calcarine sulcus	right	9	− 72	6	5.83	54
Cerebellum	right	6	− 69	− 33	5.88	46
Extrastriate cortex	right	39	− 81	9	6.79	46
Extrastriate cortex	left	− 27	− 90	3	6.51	83

This table lists the significant clusters shown by Figs. 4 and 5, with coordinates referring to the local maxima of the clusters. *k* = number of activated voxels; *x*, *y*, *z* = coordinates in MNI space; *p* < 0.05, corrected for multiple comparisons at the cluster level, using a cut-off of *p* < 0.001 at the voxel-level.
